# Demonstration of thin film pair distribution function analysis (tfPDF) for the study of local structure in amorphous and crystalline thin films

**DOI:** 10.1107/S2052252515012221

**Published:** 2015-07-05

**Authors:** Kirsten M. Ø. Jensen, Anders B. Blichfeld, Sage R. Bauers, Suzannah R. Wood, Eric Dooryhée, David C. Johnson, Bo B. Iversen, Simon J. L. Billinge

**Affiliations:** a Department of Applied Physics and Applied Mathematics, Columbia University, New York, NY 10027, USA; b Center for Materials Crystallography, Department of Chemistry and iNANO, Aarhus University, DK-8000 Aarhus C, Denmark; c Center for Sustainable Materials Chemistry, Department of Chemistry, University of Oregon, Eugene, OR 97403, USA; d National Synchrotron Light Source II, Brookhaven National Laboratory, Upton, NY 11973, USA; e Department of Condensed Matter Physics and Materials Science, Brookhaven National Laboratory, Upton, NY 11973, USA

**Keywords:** total scattering, pair distribution function analysis, thin films, framework-structured solids and amorphous materials, inorganic materials, materials modelling, nanostructure, amorphous solids

## Abstract

By means of normal-incidence, high-flux and high-energy X-rays, total scattering data for pair distribution function (PDF) analysis have been obtained from thin films (tf), suitable for local structure analysis. By using amorphous substrates as support for the films, the standard Rapid Acquisition PDF setup can be applied and the scattering signal from the film can be isolated from the total scattering data through subtraction of an independently measured background signal. No angular corrections to the data are needed, as would be the case for grazing incidence measurements. The ‘tfPDF’ method is illustrated through studies of as-deposited (*i.e.* amorphous) and crystalline FeSb_3_ films, where the local structure analysis gives insight into the stabilization of the metastable skutterudite FeSb_3_ phase. The films were prepared by depositing ultra-thin alternating layers of Fe and Sb, which interdiffuse and after annealing crystallize to form the FeSb_3_ structure. The tfPDF data show that the amorphous precursor phase consists of corner-sharing FeSb_6_ octahedra with motifs highly resembling the local structure in crystalline FeSb_3_. Analysis of the amorphous structure allows the prediction of whether the final crystalline product will form the FeSb_3_ phase with or without excess Sb present. The study thus illustrates how analysis of the local structure in amorphous precursor films can help to understand crystallization processes of metastable phases and opens for a range of new local structure studies of thin films.

## Abstract 2

It is shown how normal-incidence X-ray total scattering can be used to obtain high-quality pair distribution functions from amorphous and crystalline thin films on much thicker substrates, allowing a range of studies of the local structure in film materials.

## 1. Introduction   

Thin films are fundamental in applications from electronics to catalysis to tribology in structural materials (Nomura *et al.*, 2004 ▸; Tang *et al.*, 1989 ▸; O’Regan & Grätzel, 1991 ▸; Ginley & Bright, 2000 ▸). Even in basic science the thin film geometry allows the generation of structures that are normally metastable. For example, advanced methods in thin film preparation such a chemical vapor deposition (Hampden-Smith & Kodas, 1995 ▸; Hunt *et al.*, 1993 ▸), atomic layer deposition (George, 2010 ▸) and molecular beam epitaxy (Panish, 1980 ▸) have in recent years made it possible to prepare new, advanced functional materials with applications in, for example, thermoelectrics, semiconductors and multiferroics (Chiritescu *et al.*, 2007 ▸; Ramesh & Spaldin, 2007 ▸; Nomura *et al.*, 2004 ▸; Fortunato *et al.*, 2012 ▸). Compounds, which are unstable or metastable in the bulk state, can by means of these atomic layer engineering techniques be prepared as thin films, opening for a whole new realm of materials. When films are single crystalline, powerful methods such as coherent Bragg rod analysis (Yacoby *et al.*, 2002 ▸) and X-ray standing-wave analysis (Cowan *et al.*, 1980 ▸) can yield significant quantitative information about the ordered structure at the surface (Eom *et al.*, 1992 ▸). However, if the films are nanocrystalline or amorphous the situation becomes much more difficult. Here we describe a straightforward approach to obtain quantitative atomic pair distribution functions (PDF) from nanocrystalline and amorphous thin films yielding important local and intermediate-range structural information from films.

When preparing thin films (10–1000 nm), the precursor compounds are typically deposited on a much thicker substrate of, for example, Si, SiO_2_ or Al_2_O_3_. This sample geometry challenges the conventional methods for structural analysis using X-ray diffraction, as data collected using standard scattering configurations (*i.e.* Debye–Scherrer or Bragg–Brentano setups) are dominated by scattering from the substrate. To avoid this, grazing-incidence (GI) X-ray diffraction methods are generally applied for thin film structure analysis (Lim *et al.*, 1987 ▸). GI measurements are performed with an incident X-ray angle close to the critical angle for total external reflection, which allows the beam to illuminate as much of the thin film as possible whilst minimizing penetration of the beam into the substrate and maximizing the signal from the film. However, not only are the experiments challenging because of the very small critical angles for hard X-rays, the grazing-incidence geometry complicates analysis of the data as angular-dependent corrections for the penetration depth and the amount of illuminated sample/substrate must be made before quantitative information can be extracted. While, for example, Rietveld analysis can be performed after careful corrections and provides valuable structural insight (Quaas *et al.*, 1998 ▸; Simeone *et al.*, 2011 ▸), most of the X-ray analysis done for thin films is qualitative and used mainly for identification of crystalline phases by considering the Bragg peak position. This approach is not adequate to characterize, for example, the complex nanostructures present in modern materials, which may not possess long-range order (Billinge & Kanatzidis, 2004 ▸).

In recent years, PDF analysis has become a standard technique for characterization of local structure in bulk and nanomaterials. PDF yields structural information from amorphous, nanostructured and crystalline materials, and PDF studies have led to a breakthrough in our understanding of materials structure and reactions in materials chemistry (Billinge & Levin, 2007 ▸). In the same way, for thin films, local structural analysis could yield important information about local structure, crystallization processes and modifications from bulk structure of films. However, for PDF data corrections, grazing-incidence geometry highly complicates the data analysis (Peterson *et al.*, 2003 ▸). So far, to the best of our knowledge, PDF has therefore not been successfully applied to analysis of thin films in grazing incidence. We therefore set out to develop a method that can be used to perform quick, routine PDF analysis of thin films, here referred to as tfPDF. We show that by using high-flux, high-energy X-rays from third-eneration synchrotron sources, normal-incidence total scattering measurements can be used to extract reliable PDFs from thin films on amorphous substrates. The measurements can be made in transmission through both the sample and the substrate using the standard rapid acquisition PDF (RA-PDF) setup with a large area detector (Chupas *et al.*, 2003 ▸), making tfPDF readily available to use for a range of thin film materials.

**Figure 1:**
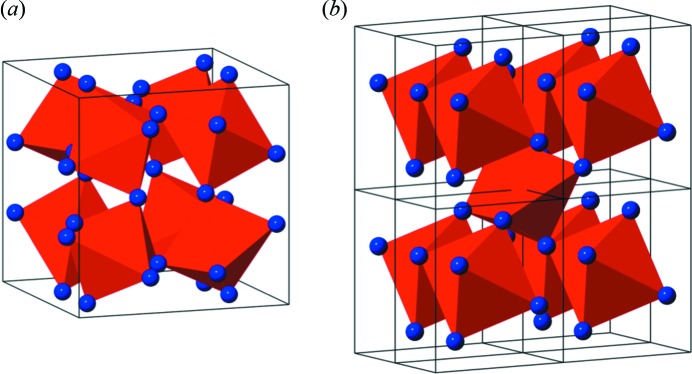
Crystal structure of (*a*) FeSb_3_ and (*b*) FeSb_2_ (eight unit cells). The red polyhedra show FeSb_6_ octahedra, with Sb marked as blue spheres in the corners.

Here, we have investigated amorphous and crystalline FeSb_*x*_ films to illustrate the feasibility of tfPDF. Deposition of alternating ultra-thin Fe and Sb layers on a flat substrate gives an amorphous film, which upon annealing crystallizes to form FeSb_2_ or FeSb_3_, depending on the thickness of the alternating Fe/Sb layers as described by Williams *et al.* (2001 ▸). The FeSb_3_ skutterudite structure is metastable, and consists of corner-sharing FeSb_6_ octahedra only (Fig. 1*a* ▸), whereas the thermodynamically stable FeSb_2_ marcasite structure has both corner- and edge-sharing octahedra (Hornbostel *et al.*, 1997 ▸) (Fig. 1*b* ▸). We set out to use tfPDF to study the local structure in the as-deposited films that leads to the metastable phase FeSb_3_, to investigate the diffusion between the Fe/Sb layers, and the relation between the precursor layering and the final crystalline product. Our studies give new insight into the crystallization of the metastable FeSb_3_ phase and open for a range of new investigations of film materials.

## 2. Experimental details   

### 2.1. Preparation of FeSb_*x*_   

The FeSb_3_ samples were synthesized using layered deposition as described in detail elsewhere (Williams *et al.*, 2001 ▸). The Sb and Fe precursors were deposited on 170 µm-thick amorphous borosilicate glass slips using the modulated elemental reactant (MER) synthesis method on a custom-built deposition system (Harris *et al.*, 2005 ▸). Antimony was deposited from a Knudsen effusion cell whereas an iron source was evaporated using an electron gun. A pressure below 5 × 10^−7^ torr was maintained during deposition. Deposition rates were monitored from quartz crystal microbalances and shutters installed above each source were sequentially opened and closed to achieve a layered precursor of the desired thickness. The deposition parameters were calibrated to allow for targeted composition ratios and bilayer thicknesses between Fe and Sb. After precursor layer deposition, the films were annealed in nitrogen for 30 min at 473 K. Compositional data were obtained with an electron probe microanalyzer, using a thin-film technique described previously (Phung *et al.*, 2008 ▸). Two sets of samples were prepared with varying Fe/Sb ratio, as listed in Table 1 ▸. The Fe layers, whose calibrated thickness was 1.0–1.1 Å, are believed to consist of a Fe monolayer covering the much thicker Sb layers. The as-deposited samples are marked A for amorphous (*i.e.* sample 1A and 2A) whereas annealed samples are marked C for crystalline (*i.e.* 1C and 2C).

### 2.2. tfPDF measurements   

**Figure 2:**
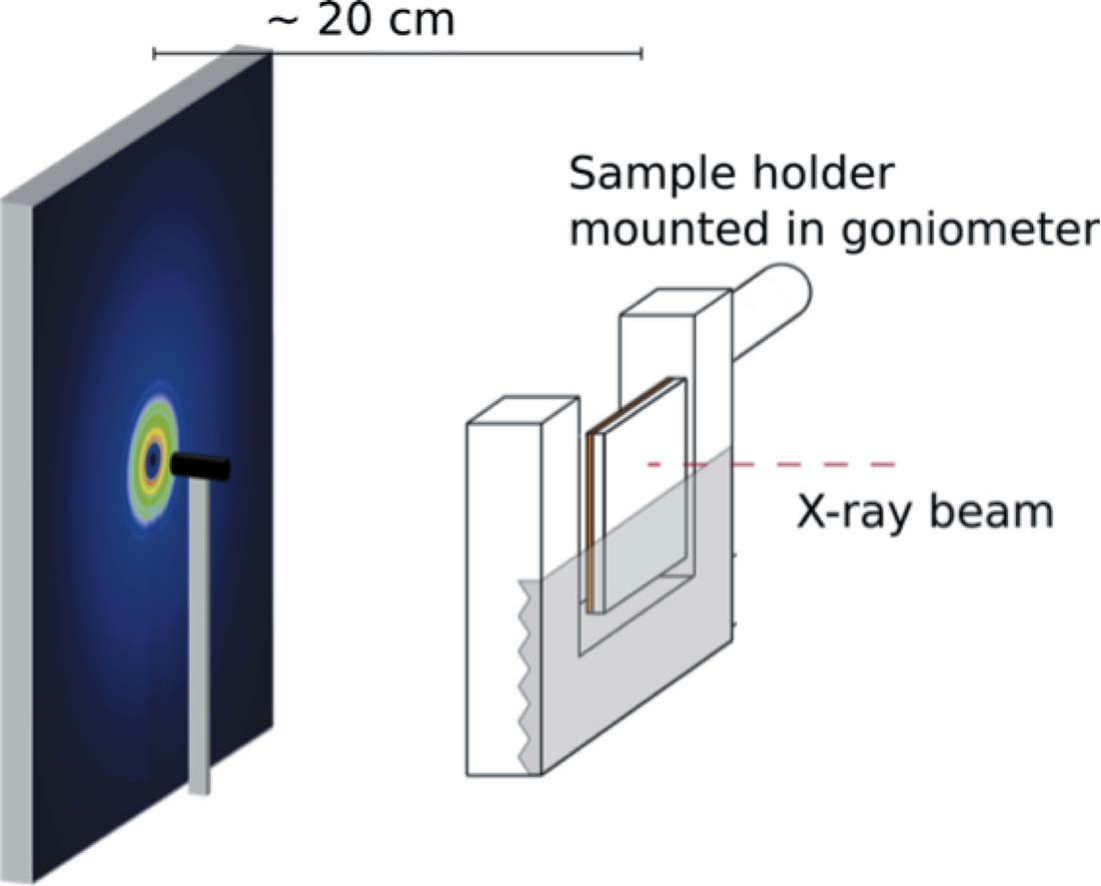
Setup used for tfPDF measurements. The X-ray beam hit the substrate before the film.

Fig. 2 ▸ shows the setup used for normal-incidence thin film PDF measurements. The films are mounted perpendicular to the beam in a simple sample holder for flat plate samples, using Kapton tape to hold the film and substrate in place. The holder is mounted and centered in the goniometer so that the beam passes through the substrate before hitting the thin film.

Data collection was carried out at the XPD beamline (X-ray Powder Diffraction, ID28) at the NSLS-II synchrotron, Brookhaven National Laboratory, USA, with a photon wavelength of 0.235 Å and a Perkin Elmer amorphous silicon detector, measuring 40 cm by 40 cm, *i.e.* in a setup similar to the usual RA-PDF geometry, making the experiments especially straightforward (Chupas *et al.*, 2003 ▸). In addition to the thin films, the scattering pattern from a clean substrate of the same material was measured, allowing background subtraction to be carried out. This approach has not been possible before because of the very low signal–background ratio in the data from a thin film sample. However, through the use of the high fluxes of hard X-rays available at modern synchrotrons, and use of the latest data reduction methods that allow very dilute signals to be successfully separated from large host signals (Terban *et al.*, 2015 ▸), we show that this approach is now possible.

Calibration of detector distance and beam center was carried out using a standard Ni sample on Kapton tape, mounted in the sample holder. Data collection took 15 min for each sample, and was made with correction for the dark-current signal. Total scattering data were also measured for reference samples of powders of amorphous and crystalline FeSb_3_ packed in a Kapton capillary as described in the supporting information.

### 2.3. Data analysis   

The PDFs were obtained from the two-dimensional data using *SrXgui* (Yang *et al.*, 2014 ▸) and *PDFgetX3* (Juhás *et al.*, 2013 ▸) in *xPDFsuite* (Yang *et al.*, 2015 ▸) as described below, with *Q*_min_ = 0.8 Å^−1^, *Q*_max_ = 17.5 Å^−1^, *Q*_max-instrument_ = 17.5 Å^−1^ and *r*_poly_ = 0.9 Å. Modeling of the PDFs was done using *PDFgui* (Farrow *et al.*, 2007 ▸), where the FeSb_3_ structure was refined in space group 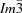 (Hornbostel *et al.*, 1997 ▸), FeSb_2_ in space group *Pnnm* and Sb in space group 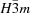. For each phase, a scale factor was refined along with unit-cell parameters and symmetry-allowed atomic positions. Isotropic Debye–Waller factors were also refined for Fe and Sb in each phase and correlated motion was taken into account by including the *delta2* parameter in the model. The coherence lengths of the crystalline phases were modeled by applying a spherical envelope to the model after taking instrumental dampening into account by modeling of a bulk Ni standard.

## 3. Results and discussion   

### 3.1. Obtaining the tfPDF: amorphous and crystalline FeSb_3_ films   

**Figure 3:**
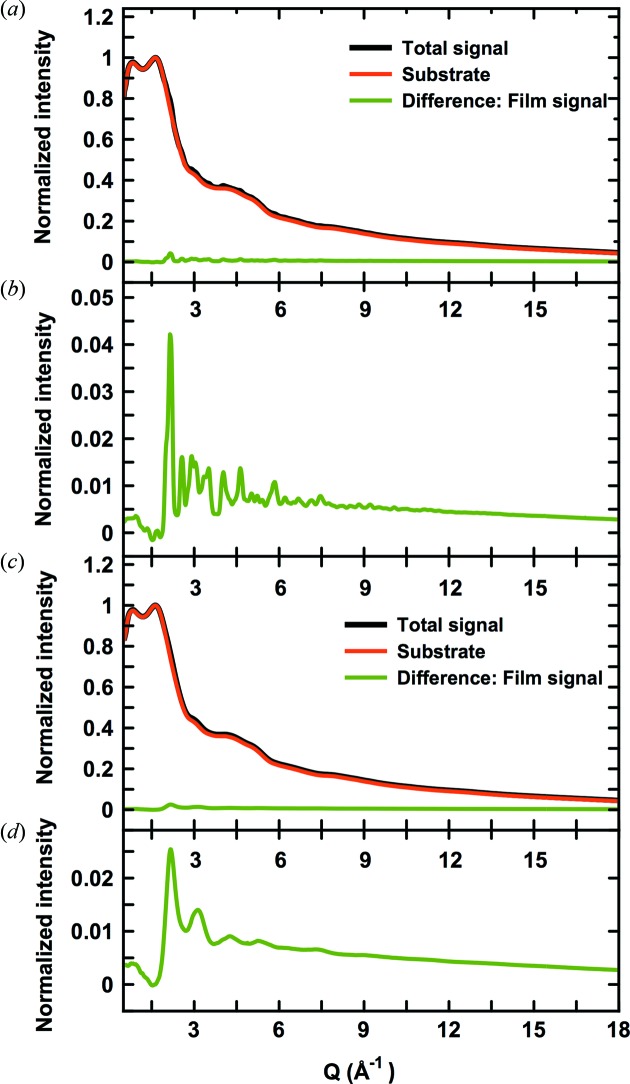
(*a*) Normalized data collected for sample 1C (black) and a clean substrate (red). The difference curve is shown in green and is plotted on an expanded scale in (*b*). (*c*) Normalized data collected for sample 1A (black) and the clean substrate (red), and difference between the two (green), also shown on an expanded scale in (*d*).

We firstly illustrate that reliable PDFs can be obtained from thin films on amorphous substrates, using the data obtained for sample 1A (amorphous) and sample 1C (crystalline) as an example. Fig. 3(*a*) ▸ (black line) shows the total X-ray scattering pattern from sample 1C, *i.e.* a 360 nm thin crystalline FeSb_*x*_ film. The thickness of the borosilicate substrate was 170 µm and thus, at normal incidence, the irradiated FeSb_*x*_ film only corresponds to *ca.* 0.21% by volume of the total sample in the X-ray beam. Accordingly, the scattering pattern shows only a very weak signal from the crystalline film while the majority of the scattered intensity is from amorphous borosilicate. In order to isolate the contribution from the FeSb_*x*_ film, the substrate contribution was determined by measuring the scattering pattern from a clean substrate, shown by the red line in Fig. 3(*a*) ▸. The Bragg peaks from the film are barely visible on top of the large substrate contribution, but become clearer after subtracting the background signal as shown in the difference between the two signals, plotted as the green curve in Fig. 3(*a*) ▸. As shown on the expanded scale in Fig. 3(*b*) ▸, Bragg peaks from crystalline FeSb_3_ are visible in the difference curve to *Q*-values at *ca.* 10 Å^−1^.

The scattering pattern from the amorphous precursor to the crystalline film is plotted in Figs. 3(*c*)–3(*d*) ▸, again showing the total signal including the background contribution [Fig. 3(*c*) ▸], as well as the weak signal from the amorphous FeSb_3_ precursor [Fig. 3(*d*) ▸]. Here, only diffuse scattering features from the amorphous film are present, but, despite this, background subtraction was still sufficient to isolate the broad peaks from the Fe/Sb signal.

PDFs from the total scattering data were obtained using *PDFgetX3* in *xPDFsuite* (Yang *et al.*, 2015 ▸). The program uses an *ad hoc* data reduction algorithm, making fast, reliable data processing possible, as individual corrections for, for example, Compton scattering and fluorescence are not needed. Instead, corrections for all long-wavelength effects in the total scattering signal are accounted for by polynomial fitting as described in detail by Juhas *et al.* (2013 ▸). This approach to data analysis makes *PDFgetX3* very well suited for data where background scattering constitutes the majority of the total signal as was previously shown for nanoparticles in very dilute systems (Terban *et al.*, 2015 ▸). Apart from correcting for the physical effects as mentioned above, the polynomial fitting applied in *PDFgetX3* can eliminate small differences between the measured background (in this case the clean substrate) and the background contribution in the sample pattern, if they are sufficiently low frequency oscillations. For standard PDF samples, these effects are on a much smaller scale than the actual signal in question and do not pose any problems in the resulting PDF. However, for small signals, such as from thin films on thick substrates, deviations such as these can be on the same scale or larger than the signal from the sample and dominate the signal after taking the difference. The *PDFgetX3* algorithm proves to be a powerful method to make these corrections that are crucial to obtain a reliable PDF from the film.

**Figure 4:**
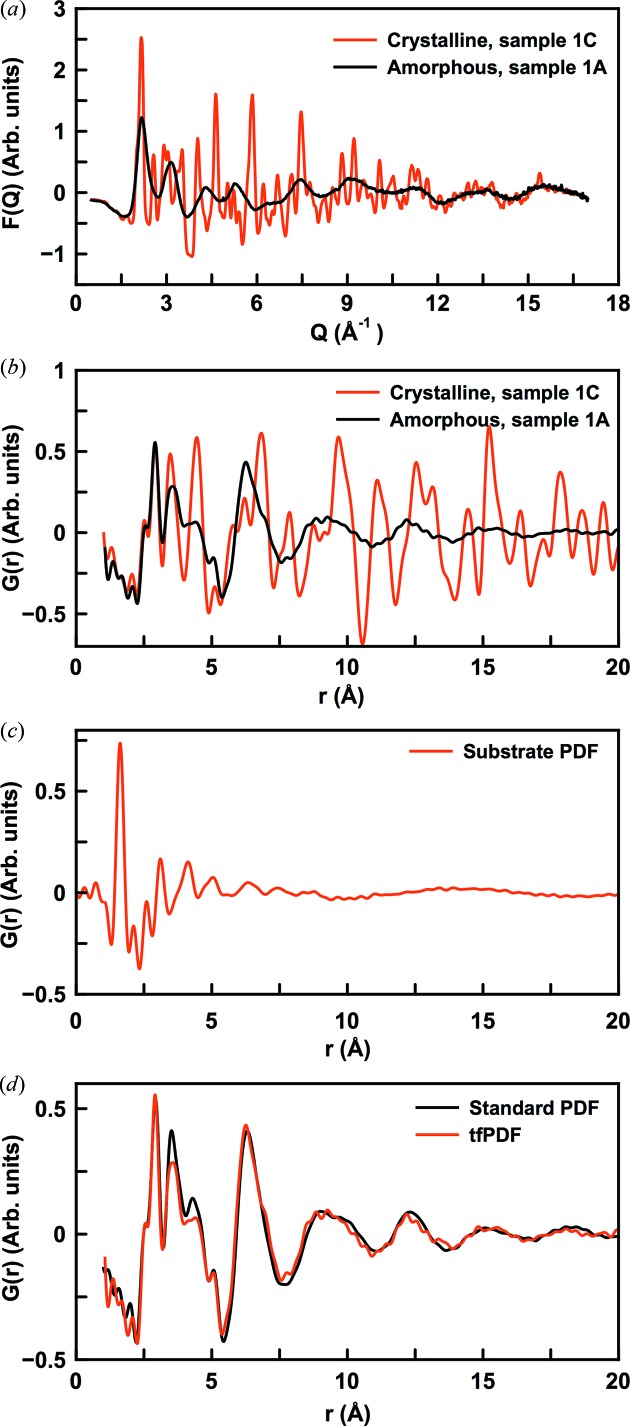
(*a*) Reduced total scattering function *F*(*Q*) for samples 1A (black) and 1C (red). (*b*) Reduced pair distribution function *G*(*r*) for 1A (black) and 1C (red). (*c*) *G*(*r*) obtained for clean substrate. (*d*) Comparison between the tfPDF for sample 1A and a powder sample of similar composition, where the data were obtained for a sample measured in a standard capillary.

The corrected, reduced total scattering functions 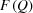 = 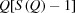 are shown in Fig. 4(*a*) ▸, for the crystalline and amorphous FeSb_3_ thin films. The substrate contribution was subtracted in *Q*-space and *F*(*Q*) thus represents the signal just from the film. Clear signals with a very low noise level even at relatively high *Q*-values are seen for both the crystalline and amorphous samples. The good data quality leads to high-quality PDFs for both the amorphous and crystalline films as shown in Fig. 4(*b*) ▸, which were obtained by Fourier transforming the *Q*-range from 0.8 to 17.5 Å^−1^. The PDF arising from a clean substrate is seen in Fig. 4(*c*) ▸. Here, a very intense peak is observed at *ca.* 1.7 Å, corresponding to the Si—O bond distance in the borosilicate glass. Inadequate background subtraction would lead to a peak at this position in the final PDF (or a negative peak when over-subtracting), but no such features are seen in Fig. 4(*b*) ▸. Minor ripples are observed which may arise from small difference between the substrates, but these are easily distinguished from the film signal.

Fig. 4(*d*) ▸ compares the tfPDF for the amorphous FeSb_3_ samples obtained from a similar sample, measured in a standard PDF setup as described in the supporting information. Clearly, the tfPDF reproduces the features from the high-quality capillary PDFs, showing that reliable PDFs are being obtained even from the 360 nm-thick thin films. The tfPDF has a higher noise level than that from the capillary data, but the structural features can easily be distinguished. Minor differences between peak intensities are observed in the 3–5 Å range, but these may be real, due to differences in Fe/Sb composition.

### 3.2. Structures in the FeSb_*x*_ system: sample 1   

**Figure 5:**
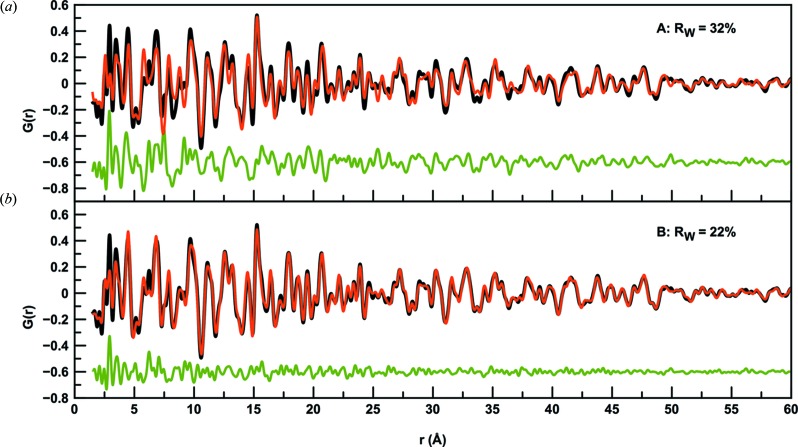
Fits (red) to the experimental PDF from sample 1C (black). The green line shows the difference curve. (*a*) Only FeSb_3_ included in the model. (*b*) FeSb_3_ and crystalline Sb included in the model.

After having established the reliability of the tfPDFs by comparison with the PDF from a capillary setup, structural information can be extracted from the data. Firstly, we analyse the tfPDFs obtained from sample 1C, *i.e.* the annealed film discussed above. Fig. 5(*a*) ▸ shows a fit of the FeSb_3_ phase to the PDF from the crystalline film. The fit gives a *R*_W_ value of 32%, showing large discrepancies between the model and data. By including crystalline Sb in the model, the *R*_w_ value is reduced to 22% and, as can be seen in Fig. 5(*b*) ▸, the model now agrees well with the experimental PDF in the high-*r*-range. The refined parameters are given in Table 2 ▸. The fit shows that the crystalline fraction of the sample contains 73% FeSb_3_ and 27% of elemental antimony. However, Fig. 5(*b*) ▸ also illustrates differences between the experimental and calculated PDF in the low-*r* region. In particular, the high intensity of the peak at 2.9 Å is not fitted well, and smaller disagreements are also seen up to *ca*. 7 Å. Considering the structure of the Sb, the peak at 2.9 Å corresponds to the shortest Sb—Sb distance as illustrated in the supporting information. The PDF thus indicates that, apart from crystalline FeSb_3_ and Sb included in the model, a fraction of amorphous Sb with only short-range order is also present in the sample. This agrees well with the elemental composition: in the total sample, the Fe/Sb ratio is 0.21 whereas in the model including only the crystalline phases this ratio is *ca*. 0.30. Neither *I*(*Q*) or *G*(*r*) showed any signs of significant texture effects in the sample and preferred orientation is not believed to contribute significantly to the misfit in the local structure.

The observed range of structural coherence, modeled using a spherical particle envelope function, of the crystalline Sb component and the FeSb_3_ phases refine to 11 and 15 nm, respectively. For the PDF to yield quantitatively reliable structures, the sample must be scattering isotropically, which is typically the case for a fine-grained powder and for nanocrystalline samples, and we have assumed that the thin film is also isotropic at the nanoscale. Based on the quality of the fits to the data, this seems to be true: there is no pronounced crystalline texture that results in some Bragg and PDF peaks being anomalously intense and others anomalously weak. However, care should be taken in general in a thin film where the anisotropy of the sample geometry may result in anisotropy in the film structure. In our tfPDF measurement geometry, the scattering vector lies predominantly in the plane of the film and so the structure is being probed predominantly in this direction (the RA-PDF experiment is not carried out in symmetric transmission so the scattering vector is not perfectly in the film plane). The measured PDF will then be a superposition of all the structural variants that exist as a function of film thickness, and anisotropies in things such as structural coherence will be sampled in a complicated way, with a greater contribution from the out-of-plane behavior in the high-angle, high-*Q* region. These effects may be deconvoluted somewhat by taking multiple measurements at different incident angles with respect to the film, though this was not done in the present case. For example, we note that the observed structural coherence of 10–15 nm is five times larger than the separation of the initial amorphous Fe/Sb layers, which alternated at *ca*. 20 Å. Williams *et al.* (2001 ▸) report that the layered structure is preserved in the amorphous phase. Our result would suggest that the layering is largely removed after annealing despite the remaining amorphous Sb component. However, some persistent layering cannot be ruled out from our current dataset for this reason.

**Figure 6:**
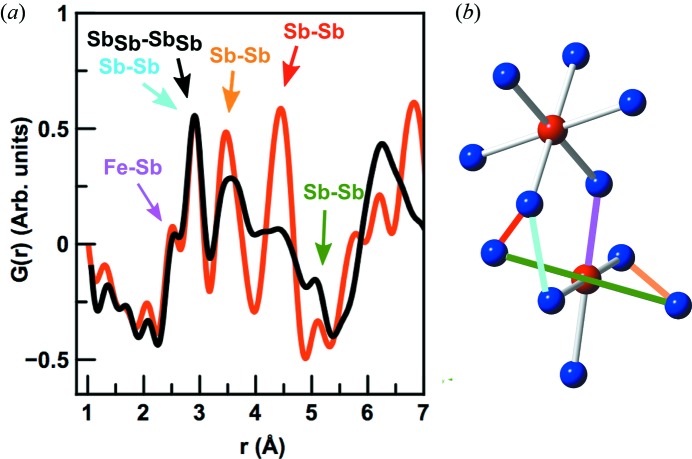
(*a*) Comparison between the PDF obtained from sample 1A (black) and sample 1C (red). Pairs contributing to the low-*r* region are indicated with arrows, and color coded with the bond illustrated in (*b*) showing a cut-out from the FeSb_3_ unit cell, with corner-sharing FeSb_6_ octahedra. Iron is shown in red and antimony in blue.

Having analysed the structure of the crystalline 1C film, we can now use the structure models to gain a better understanding of the atomic arrangement in the as-deposited precursor film, *i.e.* 1A. Fig. 6(*a*) ▸ shows a comparison between the low-*r* regions of the tfPDFs from both films. Interestingly, the local structure of the amorphous film is closely related to the crystalline structure as the first four main peaks overlap. By considering the atomic pairs leading to the peaks in the crystalline structure, we can identify the local structural motifs in the amorphous film. As seen in Fig. 1(*a*) ▸, the FeSb_3_ structure consists of corner-sharing FeSb_6_ octahedra, making up the full skutterudite lattice. A cutout of the FeSb_3_ unit cell is shown in Fig. 6(*b*) ▸ with selected interatomic distances marked and tabulated in the supporting information. The nearest-neighbour Fe—Sb distance in FeSb_3_ is *ca.* 2.6 Å [marked in purple in Fig. 6(*b*) ▸] which is seen as a clear peak in the PDFs from both the crystalline and amorphous phases. After deposition of the individual Fe/Sb layers, the metals thus immediately diffuse at room temperature to form an alloyed, amorphous structure between the Fe/Sb layers rather than staying as separate phases. The nearest intra-octahedral Sb—Sb distances in the crystalline FeSb_3_ structure arising from the edge length in the FeSb_6_ octahedra make up the broad peak centered at 3.5 Å, marked in orange in Fig. 6(*b*) ▸. Again, this peak can clearly be found in the PDF from the amorphous sample, largely overlapping with that from the crystalline PDF. The longest Sb—Sb distance in the FeSb_6_ octahedra is at 5.1 Å (marked in green), where a small peak can also be identified, thus illustrating how all intra-octahedral distances can be found in the PDF from the as-deposited sample.

The intense PDF peak at 2.9 Å originates mainly from the shortest Sb—Sb distances in the elemental crystalline Sb phase as described above, marked with black in Fig. 6(*a*) ▸. This peak is clearly present in the amorphous phase, so apart from the interdiffused Fe—Sb structures the amorphous phase appears to also contain a fraction of amorphous Sb not atomically coordinated to Fe. In crystalline Sb, the second nearest-neighbor Sb—Sb distance is at 3.34 Å, and, from the theoretical PDF from Sb metal, this peak should have *ca*. 80% of the intensity of the peak at 2.9 Å. However, this peak is not clear in the PDF from sample 1A, indicating that the local structure of the amorphous Sb fraction in the as-deposited sample does not resemble that of crystalline Sb, where the atoms are arranged in layers of six-membered rings.

As indicated in Fig. 6(*a*) ▸ and explained in more detail in the supporting information, the PDF peak at 4.3 Å in crystalline FeSb_3_ arises from a number of inter-octahedral correlations, one shown in red in Fig. 6(*b*) ▸. A broad peak in the same region is seen in the PDF from the amorphous phase. In crystalline FeSb_3_, an inter-octahedral Sb—Sb distance marked in cyan in Fig. 6 ▸ furthermore gives rise to a weak peak at *ca*. 2.9 Å. However, compared with the Sb—Sb distance in crystalline Sb metal, this is only a minor contribution to the total PDF of the crystalline sample, and we cannot distinguish this from the elemental Sb—Sb peak in sample 1A.

The observation of the existence of FeSb_6_ octahedra as well as amorphous Sb points to a structure where amorphous Sb structures with only short-range order coexist with disordered, corner-sharing FeSb_6_ octahedra. The local structure of the amorphous precursor before thermal annealing thus highly resembles that of the metastable FeSb_3_ phase, explaining the possibility to synthesize it from the layered precursors.

### 3.3. Structures in the FeSb_*x*_ system: sample 2   

**Figure 7:**
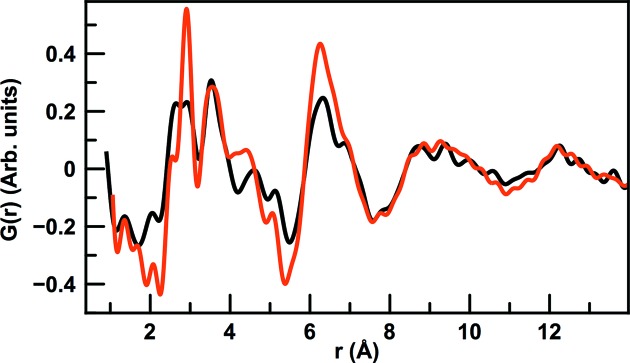
Comparison between the PDFs from sample 1A (red) and 2A (black).

Sample 2 was prepared with slightly lower antimony content than sample 1. Fig. 7 ▸ compares the PDFs from sample 1A and 2A, *i.e.* the two amorphous samples. While some of the peaks discussed above are also evident in the 2A PDF, we also observe clear differences in the local structure. The first peak at 2.6 Å again corresponds to the Fe—Sb distance in FeSb_6_ polyhedra and peaks from the Sb—Sb distances in the octahedra (at *ca*. 3.5 Å and 5.1 Å; see Fig. 6 ▸) are also seen. Furthermore, a contribution at 2.9 Å is also present, corresponding to the first Sb—Sb distance in metallic Sb as discussed above. However, compared with 1A, this peak is much less dominant, indicating a smaller contribution of Sb not coordinated to Fe. This agrees with the measured compositions, where the Fe/Sb ratio is 0.33. We also see a difference in the width and position of the peak at *ca*. 4.6 Å, which we above ascribed to correlations between the individual octahedral. Possibly, the lower Sb content changes the local structure around the octahedra units.

**Figure 8:**
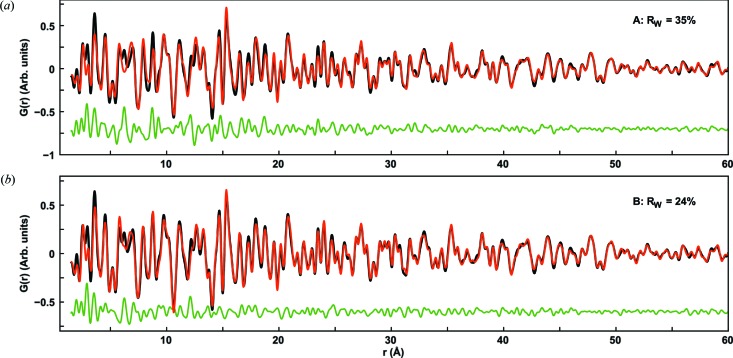
(*a*) Fit of FeSb_3_ and Sb to the PDF from sample 2C. (*b*) Fit of FeSb_3_ and FeSb_2_ to the PDF from sample 2C. The experimental PDF is shown in black, the fit in red and the difference in green.

The appearance of the PDF from sample 2A indicates that the corresponding annealed sample 2C will contain a smaller Sb content than sample 1C. This is confirmed when modeling the PDF, as a two-phase fit with FeSb_3_ and Sb results in crystalline phase fractions of 99% and 1%, respectively, thus effectively suppressing the Sb phase completely. However, interestingly, the fit of the FeSb_3_ phase is still of poor quality, giving *R*_W_ = 35% and large deviations as seen in Fig. 8(*a*) ▸. When introducing the thermodynamic phase in the phase diagram, FeSb_2_, the fit improves considerably (Fig. 8*b* ▸) giving *R*_W_ of 25%. The refined parameters for this fit are given in Table 3 ▸. The refined phase fractions are 73% FeSb_3_ and 27% FeSb_2,_ with the coherence length in the FeSb_2_ phase being *ca*. 7 nm. The coherence length of the FeSb_3_ phase refines to *ca*. 50 nm, which is well above the reliable limit for size determination but indicates that this component forms very large crystallites. The lower Sb content in the precursor thus has two effects: suppression of crystalline Sb while forming a phase mixture between the thermodynamic FeSb_2_ phase and the metastable FeSb_3_ as well as allowing the FeSb_3_ to grow into a bulk phase.

## 4. Conclusion   

PDFs have been obtained from supported thin film samples, using normal-incidence X-ray diffraction measurements in a standard RA-PDF setup. The use of high-flux, high-energy X-rays and careful background subtraction in *Q*-space make it possible to obtain a clear scattering signal from amorphous, nanocrystalline and polycrystalline films down to a thickness of at least a few hundred nanometers, which by use of *xPDFsuite* and *PDFgetX3* can be Fourier transformed into PDFs of high quality (Yang *et al.*, 2015 ▸).

All films studied were deposited on amorphous substrates, as this allows for simple subtraction of the substrate scattering signal without the need to mask intense, orientation-dependent scattering signals from single-crystal substrates, *e.g.* silicon wafers. No angular-dependent corrections are needed, as would be the case for grazing-incidence measurements. By use of *PDFgetX3*, where *ad hoc* corrections for fluorescence, Compton scattering and any other non structural effects are carried out, PDFs can be obtained quickly in a robust manner. The thin films that have been studied here are all *ca*. 360 nm thick, but PDFs from even thinner films may also be obtained, as long as background subtraction of the substrate signal is performed.

The characterization of thin film has so far been limited by the need for grazing-incidence techniques, which is still to be reported for PDF analysis. In some cases, the film can be isolated from the substrate and standard characterization techniques can be used, but most often this is not possible due to the small mass of sample present as film. tfPDF thus opens the way for many new possibilities in materials characterization for thin films. As shown in the case of the FeSb_*x*_ samples, tfPDF can be used to understand the relation between the local structure in amorphous films and the final crystalline product, which will help chemists in controlled synthesis of new, advanced materials, in thin film form. We now plan to use tfPDF for *in situ* studies, where a much deeper understanding of processes like this (diffusion, nucleation, crystallization) can be understood. While the time resolution is limited by longer counting times required for the small amount of sample present in the beam, the new high-flux beamlines at third-generation synchrotrons suitable for PDF analysis will allow these studies to be feasible.

## 5. Related literature   

The following reference is mentioned in the supporting information: Hammersley *et al.* (1996) ▸. The supporting information includes a description of the sample preparation for capillary samples, illustration of the Sb metallic structure and a histogram of interatomic distances in FeSb_3_.

## Supplementary Material

Description of the sample preparation for capillary samples, illustration of the Sb metallic structure and a histogram of interatomic distances in FeSb3. DOI: 10.1107/S2052252515012221/yu5008sup1.pdf

## Figures and Tables

**Table 1:** Sample list

Sample name	Structure	Layer thickness	Fe/Sb ratio	Film thickness
1A	As-deposited, amorphous	Fe: 1.1	0.21	3600
		Sb: 17.0		
1C	Annealed, crystalline	Fe: 1.1	0.21	3600
		Sb: 17.0		
2A	As-deposited, amorphous	Fe: 1.0	0.33	3600
		Sb: 12.0		
2C	Annealed, crystalline	Fe: 1.0	0.33	3600
		Sb: 12.0		

**Table 2:** Refined parameters for the crystalline

*R*_W_	23.7%
Weight percent, FeSb_3_	72.7%
*a*, FeSb_3_	9.185
Crystallite size, FeSb_3_	15.1 nm
*y*_Sb_, FeSb_3_	0.337
*z*_Sb_, FeSb_3_	0.159
*U*_iso,Fe_, FeSb_3_	0.0174 ^2^
*U*_iso,Sb_, FeSb_3_	0.0171 ^2^
Weight percent, crystalline Sb	27.3%
*a*, Sb	4.299
*c*, Sb	11.291
Crystallite size, Sb	11.2 nm
*z*, Sb	0.767
*U*_iso,Sb_, Sb	0.0098 ^2^
delta2†	4.08

† The delta2 parameters for the two phases, expressing correlated motion, were constrained to the same value.

**Table 3:** Refined parameters for modeling of sample 2C

*R*_W_	23.8%
Phase fraction, FeSb_3_	73.2%
*a*, FeSb_3_	9.219
Particle diameter, FeSb_3_	47 nm
*y*_Sb_, FeSb_3_	0.334
*z*_Sb_, FeSb_3_	0.158
*U*_iso,Fe_, FeSb_3_	0.0073 ^2^
*U*_iso,Sb_, FeSb_3_	0.0100 ^2^
Phase fraction, FeSb_2_	26.8%
*a*, FeSb_2_	5.836
*b*, FeSb_2_	6.572
*c*, FeSb_2_	3.221
Particle diameter, FeSb_2_	7.8 nm
*x*_Sb_, FeSb_3_	0.187
*y*_Sb_, FeSb_3_	0.357
*z*_Sb_, FeSb_3_	0.030
*u*_iso,Fe_, FeSb_3_	0.0086 ^2^
*u*_iso,Sb_, FeSb_3_	0.0046 ^2^
