# Supplementary material for: Demonstration of thin film pair distribution function analysis (tfPDF) for the study of local structure in amorphous and crystalline thin films
Source: IUCrJ. 2015 Jul 5;2(Pt 5):481–9. doi: 10.1107/S2052252515012221 (PMC4547816; doi:10.1107/S2052252515012221)
Supplement: Supplementary file 1 [file m-02-00481-sup1.pdf]

IUCrJ

Volume 2 (2015)

Supporting information for article:

Demonstration of thin film pair distribution function analysis (tfPDF) for the study of local structure in amorphous and crystalline thin films

Kirsten M. Ø. Jensen, Anders B. Blichfeld, Sage R. Bauers, Suzannah R. Wood, Eric Dooryhee, David C. Johnson, Bo B. Iversen and Simon J. L. Billinge

S1. Preparation and measurements of samples in capillaries

The Fe/Sb precursors were deposited on (100) Si wafers coated in PMMA. The films were then floated off of the support wafer by dissolving the PMMA in acetone, washed to remove excess PMMA, and collected on a Teflon filter. This resulted in thin metallic flakes, which were packed into a 1.0mm kapton capillary. The total scattering data collection for the capillary samples was done at X17A at the NSLS synchrotron. Total scattering data were acquired with an X-ray wavelength of 0.18597 Å using a Perkin Elmer amorphous silicon detector measuring 40 by 40 cm^2. The 2D data were integrated using *Fit2D*[1] and PDFs were obtained with *PDFgetX3*[2] using a Q_{min} of 0.8 $Å^{-1}$, Q_{max} of 17.5 $Å^{-1}$, and an *rpoly* of 0.9.

S2. Crystal structure of elemental antimony

The crystal structure of elemental antimony is illustrated in Figure S1, showing 4 unit cells. The structure consists of layers of 6-membered rings, connected in a zig-zag pattern.

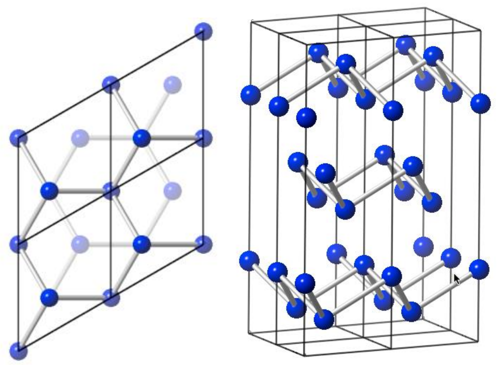

Figure S1 4 unit cells of the Sb structure (*R-3m)*. The left figure shows the top view (along *c*) while the right figure show the side view, illustrating layering in the *ab*-plane.

S3. Histogram of interatomic distances in $FeSb_3$

Figure S2 show the histogram of interatomic distances in the $FeSb_3$ system scaled after number of correlations (blue) and number of electrons (i.e. X-ray scattering power, red). The interatomic distances are given in Table S1. As described in the main text, the peaks at 2.6 Å 3.4 Å, 3.8 Å, 4.2 Å and 5.1 Å arise from intra-octahedra distances. The small peak at 2.9 Å originates from the shortest interoctahedral Sb-Sb distance. Furthermore, all peaks between 4.1 Å and 5 Å arise from inter-octahedra distances, where one is illustrated in the main text.

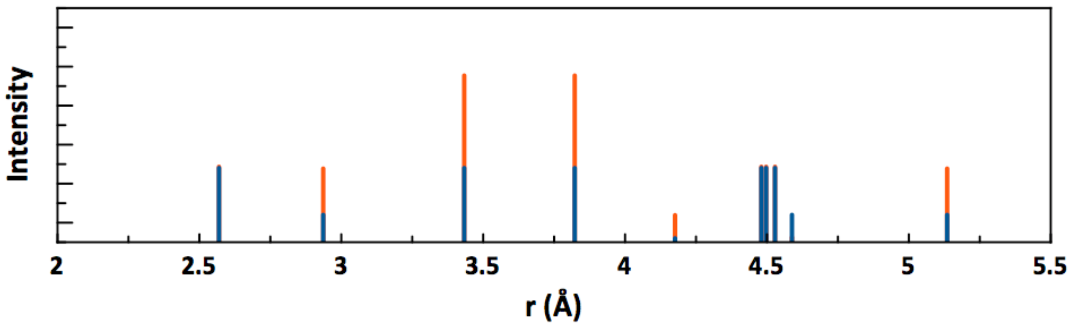

Figure S2 Histogram of interatomic distances between 0-6 Å in the $FeSb_3$ structure. The blue bars are scaled by number of pairs, while the red bars are scaled by the number of electrons involved (proportional to the X-ray scattering power).

Table S1 List of interatomic distances in crystalline $FeSb_3$ from 0-6 Å,

**Peak position (Å)**	**Pair**	**Type**	**Number of correlations**
2.569	Fe-Sb	Intra-octahedral	48
2.936	Sb-Sb	Intra-octahedral	24
3.433	Sb-Sb	Intra-octahedral	48
3.823	Sb-Sb	Intra-octahedral	48
4.176	Sb-Sb	Inter-octahedral	12
4.480	Fe-Sb	Inter-octahedral	48
4.497	Fe-Sb	Inter-octahedral	48
4.528	Fe-Sb	Inter-octahedral	48
4.588	Fe-Fe	Inter-octahedral	24
5.135	Sb-Sb	Intra-octahedral	24

References

1. Hammersley, A. P.; Svensson, S. O.; Hanfland, M.; Fitch, A. N.; Hausermann, D. *High Pressure Res.* 1996, 14, 235-248.

2. Juhas, P.; Davis, T.; Farrow, C. L.; Billinge, S. J. L. *J. Appl. Crystallogr.* 2013, 46, 560-566.
